# QuickStats

**Published:** 2015-05-08

**Authors:** FROM THE NATIONAL CENTER FOR HEALTH STATISTICS

**Figure:**
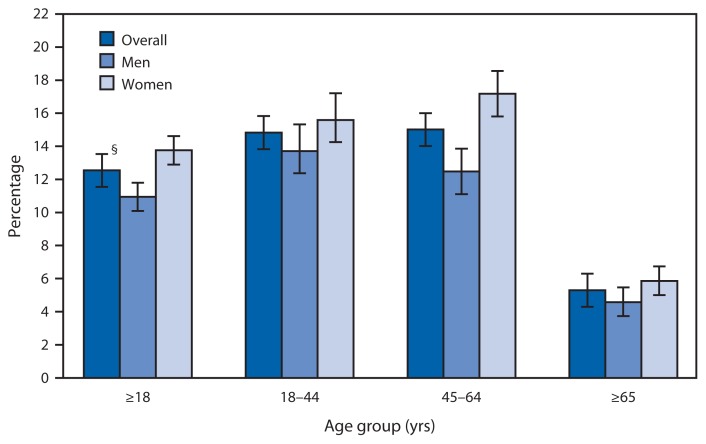
Percentage of Adults Who Did Not Take Medication as Prescribed to Save Money,^*^ Among Those Prescribed Medication During the Preceding 12 Months, by Sex and Age Group — National Health Interview Survey,^†^ United States, 2013 ^*^ Based on a positive response to any of the following three survey questions: “You skipped medication doses to save money; you took less medicine to save money; or you delayed filling a prescription to save money.” In 2013, these questions were asked to those who reported having been prescribed medication by a doctor or other health professional during the preceding 12 months, and referred to actions to save money during the preceding 12 months. ^†^ Estimates are based on household interviews of a sample of the civilian noninstitutionalized U.S. population and are derived from the National Health Interview Survey Sample Adult component. ^§^ 95% confidence interval.

In 2013, 12.5% of adults overall who were prescribed medication by a doctor or other health professional did not take their medication as prescribed to save money. Adults aged ≥65 years were less likely to not take their medication as prescribed (5.3%) than those aged 18–44 years (14.8%) and those aged 45–64 years (15.0%). Women (13.8%) were more likely than men (10.9%) to not take their medication as prescribed, with the largest difference observed between women and men aged 45–64 years (17.2% compared with 12.5%).

**Source:** National Health Interview Survey, 2013. Available at http://www.cdc.gov/nchs/nhis.htm.

**Reported by:** Maria A. Villarroel, PhD, mvillarroel@cdc.gov, 301-458-4668; Robin A. Cohen, PhD.

